# Impacts of Hemodynamic Support on Ventricular Arrhythmia Ablation

**DOI:** 10.1111/jce.70396

**Published:** 2026-06-09

**Authors:** Denise Guckel, Moneeb Khalaph, Mustapha El Hamriti, Thomas Fink, Vanessa Sciacca, Maximilian Moersdorf, Guram Imnadze, Martin Braun, Angelika Costard‐Jäckle, René Schramm, Jan Gummert, Philipp Sommer, Christian Sohns

**Affiliations:** ^1^ Clinic for Electrophysiology, Herz‐ und Diabeteszentrum NRW Ruhr‐Universität Bochum Bad Oeynhausen Germany; ^2^ Clinic for Thoracic and Cardiovascular Surgery, Herz‐ und Diabeteszentrum NRW Ruhr‐Universität Bochum Bad Oeynhausen Germany

**Keywords:** ablation, heart failure, hemodynamic support, mortality, ventricular arrhythmias

## Abstract

**Background:**

Data on the efficacy of hemodynamic support (HS) for protecting ventricular arrhythmia (VA) ablation in high‐risk heart failure (HF) patients with an impaired left ventricular function and its impact on mortality is limited.

**Objectives:**

This study aimed to assess procedural efficacy, related complications, VA‐free survival, and mortality of HF patients who underwent protected VA ablation with HS.

**Methods:**

Data from consecutive HF patients undergoing VA ablation at our center between 2018 and 2024 were analyzed. HS included the treatment with an Impella, an extracorporeal membrane oxygenation (ECMO) or a left ventricular assist device (LVAD). Patients were classified based on their HF status according to current guidelines.

**Results:**

A total of 187 patients (61.5 ± 10.9 years) were included. 124/187 patients were in advanced HF (66%, 63.5 ± 8.9 years). In 68/187 patients (36%, 60.8 ± 9.8 years), HS was required. Procedural parameter did not differ between the groups except for fluoroscopy times (*p* < 0.001). Acute procedural success (*p* = 0.670) and major procedure‐related complications (*p* = 0.726) were comparable. In‐hospital mortality was higher in patients with HS (*p* < 0.001). In advanced HF patients, freedom from VA‐recurrence was lower (*p* = 0.016) and mortality higher (*p* = 0.009). HS with an ECMO and LVAD, but not with an Impella, was associated with increased VA‐recurrence (hazard ratio [HR] 3.66, confidence Interval [CI] 1.732–7.715, *p* < 0.001) and mortality (HR 3.95, CI 1.091–14.280, *p* = 0.036).

**Conclusion:**

In high‐risk HF patients, VA ablation was feasible with comparable acute success and procedural complication rates. Patients with ECMO or LVAD showed higher in‐hospital mortality and higher VA recurrence compared to Impella‐supported procedures, likely reflecting more advanced disease severity rather than differences in procedural efficacy.

**Abbreviations**
AHD: acute hemodynamic decompensation
CRT: cardiac resynchronization therapy
ECMO: extracorporeal membrane oxygenation
HF: heart failure
HS: hemodynamic support
ICD: implantable cardioverter‐defibrillator
LVAD: left ventricular assist device
LVEF: left ventricular ejection fraction
NYHA: New York heart association functional class
VA: ventricular arrhythmia

## 1 Introduction

Catheter ablation (CA) has emerged as an effective treatment strategy for ventricular arrhythmias (VA) [1, 2, 3]. High‐risk patients with advanced heart failure (HF), recurrent drug‐refractory sustained VA, and implantable cardioverter‐defibrillator (ICD) therapies are increasingly scheduled for VA ablation procedures [2, 3]. These patients are predisposed to acute hemodynamic decompensation (AHD) during the procedures [4]. Risk scores have been designed to facilitate an appropriate preprocedural risk assessment, as the integration of hemodynamic support (HS) in the event of an AHD appears to be associated with a poor prognosis [5, 6]. The pre‐emptive use of HS may enhance mapping and ablation, which may have beneficial effects on the outcome of high‐risk patients who require VA ablation [7, 8]. However, only a handful of studies have examined the safety and efficacy of different types of HS [9, 10, 11]. Given that VA frequently occur before and particularly after left ventricular assist device (LVAD) implantation, CA of drug‐refractory VA is crucial in the care of patients with advanced HF and ventricular assist devices, too [12].

There is a lack of data regarding freedom from arrhythmia recurrence and mortality of high‐risk patients who received VA ablation procedures with HS. In particular, effects of advanced HF on patients’ outcome need to be evaluated and a more detailed differentiation depending on the type of HS is required. Therefore, this study aimed to analyze acute procedural success, complications, 24‐month VA‐recurrence rates, and mortality in patients with and without advanced HF who underwent VA ablation.

## 2 Methods

In this observational study, data from 187 consecutive patients who underwent CA for scar‐related VA at our center between 2018 and 2024 were included. Among these, selected patients received preestablished HS (ECMO, Impella, LVAD) based on patient‐specific hemodynamic and clinical characteristics. All patients had ischemic or non‐ischemic HF and underwent VA ablation in the setting of structural heart disease.

**Figure 1:**
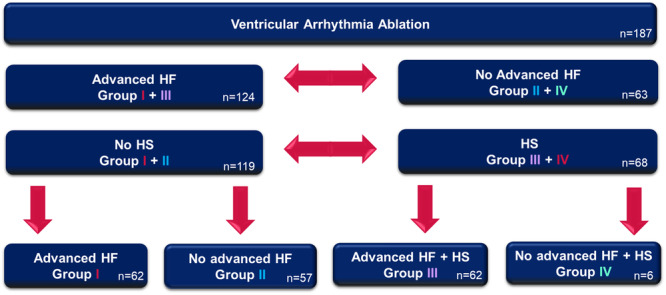
Patients. Consecutive HF patients undergoing ventricular arrhythmia ablation were divided into four groups allowing for a direct comparison of advanced HF patients (groups I + III) to patients without advanced HF (groups II + IV) and patients with HS (groups I + II) to those without (groups III + IV). Beyond that, the cohort of patients with advanced HF and HS (group III) was compared to patients with advanced HF that did not require HS (group I). HF, heart failure; HS, hemodynamic support.

Patients were classified as advanced HF‐patients adherent to the definition of the ESC Heart Failure Association (left ventricular ejection fraction [LVEF] < 30%, New York Heart Association [NYHA] functional classes **≥** III, elevated NT‐proBNP‐levels > 1.500 pg/mL) (groups I + III) [13]. These patients were compared to patients without advanced HF (LVEF > 30%, NYHA classes < III and NT‐proBNP‐level < 1.500 pg/mL) (groups II + IV). Additionally, patients who required HS (groups III + IV) were compared to those who underwent VA ablation procedures without HS (groups I + II) (Figure 1). HS was defined as the use of an Impella 2.5 (Abiomed, Inc, Massachusetts, USA), an extracorporeal membrane oxygenation (ECMO) or a LVAD (HeartMate 3, Abbott, Abbott Park, IL, USA). Data on procedural parameters, acute success and complications as well as 24‐month‐VA‐free survival and mortality was analyzed. VA‐recurrence does include VA treated by ICD therapy as well as any documented episodes of sustained VA > 30 s. All patients were continuously followed up in our outpatient clinic. All patients gave written informed consent and the study was performed in compliance with the principles outlined in the Declaration of Helsinki and approved by the Institutional Ethics Committee (Reg. No. 2019‐563).

### 2.1 HS

The study cohort included patients with HF and structural heart disease undergoing VA ablation under HS. All patients with LVAD had permanent LVAD support established prior to the ablation procedure and were referred for treatment of recurrent VA during ongoing LVAD therapy. ECMO support was initiated prior to the procedure in patients with pre‐existing hemodynamic instability or cardiogenic shock. Impella support was used in pre‐emptive high‐risk cases based on established risk stratification with the help of the PAINESD risk score (chronic obstructive pulmonary disease, age > 60 years, ischemic cardiomyopathy, New York Heart Association functional class III or IV, ejection fraction < 25%) score [4]. Systematic assessment of right ventricular (RV) function using standardized echocardiographic or invasive hemodynamic parameters is not consistently available across all patients. Right heart catheterization to determine use and type of HS devices was not routinely performed prior to the procedure. All devices were implanted prior to the ablation procedure and were not used as a rescue strategy for intraprocedural hemodynamic instability.

### 2.2 Ablation Procedure

Prior to ablation, thrombus formation was ruled out using echocardiography. ICD/CRT‐D devices were interrogated before ablation and all VA‐therapies were deactivated.

Mapping and ablation were performed under either conscious sedation or general anesthesia. During the procedure, heparin was administered to maintain an activated clotting time of ≥ 300 s. Two diagnostic catheters were introduced via the femoral veins and positioned in the coronary sinus (6Fr, Webster, Biosense Webster, Inc., Diamond Bar, CA, USA) and the right ventricle (5Fr, Webster, Biosense Webster, Inc., Diamond Bar, CA, USA). VA induction with programmed ventricular stimulation from two ventricular sites at two drive cycle lengths with up to triple ventricular extra stimulation were performed. If a sustained tolerated VA was present at the beginning of the procedure or induced, activation mapping (CARTO3, Biosense Webster, Inc., Diamond Bar, CA, USA or Ensite Precision, Abbott Lab., Chicago, IL, USA) was performed and if VA was non‐tolerated, the tachycardia was terminated by burst ventricular pacing or direct current cardioversion. For an antegrade approach, venous access was obtained via a femoral vein and a single transseptal puncture was performed under fluoroscopic guidance using a modified Brockenbrough technique and an 8.5Fr transseptal sheath (CARTO ViZiGo, Biosense Webster Inc., Diamond Bar, CA, USA or Agilis, St. Jude Medical, Inc., St. Paul, MN, USA). A retrograde access into the left ventricle was obtained via a femoral artery with insertion of a 8.5Fr sheath (SL1, St. Jude Medical, Inc., St. Paul, MN, USA), if necessary. Myocardial scar and the border zone in the left ventricle were delineated using ultra high‐density voltage mapping (aiming for > 1000 mapping points) utilizing a three‐dimensional (3D) electroanatomical mapping system and a multipolar mapping catheter (PentaRay, Biosense Webster, Inc., Diamond Bar, CA, USA or HD Grid, Abbott Lab. Chicago, IL, USA). Low‐voltage areas, suggestive for myocardial scar tissue, were defined at bipolar voltage of ≤ 1.5 mV. In addition, any local abnormal electrograms (EGMs) in the form of fractionated, late, and abnormal decrement‐evoked local ventricular EGMs unmasked during pacing with ventricular extra‐stimuli were tagged on the 3D reconstruction. After mapping, comprehensive substrate ablation was performed targeting all low‐voltage areas and abnormal EGMs. CA was conducted uniformly between different operators and in sinus rhythm or atrial/ventricular pacing with radiofrequency current using an irrigated‐tip, contact force sensing ablation catheter (ThermoCool, Biosense Webster, Inc., Diamond Bar, CA, USA or Flexability, Abbott Lab., Chicago, IL, USA). Once scar homogenization was completed, substrate modification and elimination of abnormal EGMs was verified with high‐density remapping and repeat programmed ventricular stimulation adherent to the ventricular stimulation protocol mentioned above.

Acute procedural success was defined as non‐inducibility of any sustained VA at the end of the procedure. After ablation, pericardial effusion was ruled out and patients were scheduled for regular follow‐up visits in our outpatient clinic. The ICD/CRT‐D device underwent a reset and reactivation after ablation.

### 2.3 Follow‐up

After hospital discharge, regular follow‐up visits were scheduled at our outpatient clinic including device interrogations or routine 7‐day Holter ECGs and interviews. Unscheduled visits were conducted if required. VA recurrence was judged on ECG or IEGM documentations and symptoms suggestive for arrhythmia recurrence.

### 2.4 Endpoint

We aimed to analyze the acute procedural success, complications, 24‐month VA‐recurrence and mortality rates in patients with and without advanced HF that had undergone VA ablation. The impact of different modalities of HS on individual patient outcomes was examined.

### 2.5 Data Collection

Data on the characteristics, medication, symptoms, and complications of patients were collected from their records and discharge letters. Procedural parameters and clinical aspects related to CA were obtained from ablation protocols and procedure‐related documents.

### 2.6 Statistical Analysis

Statistical analyses were performed with SPSS, version 29 (SPSS, Inc., Chicago, IL, USA). All variables were tested for normal distribution. Two‐tailed *p*‐value analyses were performed. Continuous variables between the groups were compared by employing an unpaired two‐sided Student's *t*‐test or Mann–Whitney test. Differences in continuous parameters between baseline and follow‐up were analyzed by paired Student's *t*‐test or Wilcoxon signed‐rank test. Categorical and ordinal data were examined by chi‐square, Mann–Whitney tests, or Fisher's exact tests, respectively. Event‐free survival and mortality were calculated by Kaplan–Meier analyses as time from initial VA ablation to first documented VA episode > 30 s or death within the observation period. The log‐rank test was used to assess differences in event‐free survival time between groups. A Cox proportional hazard regression model was applied to identify independent predictors (IPs) of arrhythmia recurrence and mortality. Demographic and clinical data from baseline analyses were included in univariate Cox proportional hazard regression models for the primary endpoint. Variables with an unadjusted association with VA recurrence or mortality (*p* < 0.1) were analyzed by multivariate Cox regression analysis. The cumulative incidence of VA, accounting for the competing risk of death, was assessed through Fine and Gray subdistribution hazard models using R (version 4.4.0; R Foundation for Statistical Computing, Vienna, Austria).

Data are presented as mean ± SD or percentage value unless stated otherwise. A *p*‐value ≤ 0.05 was considered statistically significant.

## 3 Results

### 3.1 Patients

A total of 187 HF patients (mean age 61.5 ± 10.9 years, 13% female) were included. One hundred twenty‐four patients fulfilling the criteria of advanced HF (66%, mean age 62.1 ± 9.2 years, 14% female; groups I + III) were compared to 63 patients with an impaired LVEF who did not meet the criteria for advanced HF (34%, mean age 60.5 ± 13.8 years, 13% female; groups II + IV). Ablation was performed with HS in 68 patients (36%, mean age 60.8 ± 9.8 years; groups III + IV) (Figure 1). Patients with temporary (ECMO, Impella) and permanent HS (LVAD) demonstrated differences in baseline characteristics and clinical profiles, reflecting distinct underlying disease trajectories. Subgroup analyses were performed to account for potential differences between HS modalities. A comparison was conducted involving data from 20 patients with ECMO (29%, mean age 59.4 ± 11.4 years, 25% female), 19 patients with Impella (28%, mean age 61.1 ± 11.2 years, 11% female), and 29 patients with LVAD support (43%, mean age 61.1 ± 7.7 years, 14% female).

### 3.2 Baseline Characteristics

#### 3.2.1 Advanced HF Versus No Advanced HF

The baseline characteristics of the 124 patients with advanced HF were significantly different from those of the 63 patients without advanced HF (Tables 1 and S1). Advanced HF patients were substantially more likely to be diagnosed with hypertension, an ischemic cardiomyopathy (ICM), high NT‐proBNP values, Amiodarone therapy, and high PAINESD scores. In the majority of patients, an ongoing AAD therapy with betablockers and amiodarone was maintained following ablation.

**Table 1:** Baseline characteristics (advanced HF vs. no advanced HF, HS vs. no HS).

Characteristics	Advanced HF (I + III) (*n* = 124)	No advanced HF (II + IV) (*n* = 63)	*p*‐value	HS (III + IV) (*n* = 68)	No HS (I + II) (*n* = 119)	*p*‐value
Age (years)	62.06 ± 9.18	60.46 ± 13.80	0.409	60.78 ± 9.77	61.94 ± 11.58	0.466
Gender, Female	17 (14%)	8 (13%)	1.000	11 (16%)	14 (12%)	0.503
ICM	79 (64%)	26 (41%)	**0.005**	44 (65%)	61 (51%)	0.092
NICM	45 (36%)	37 (59%)	**0.005**	24 (35%)	58 (49%)	0.092
NTproBNP (pg/mL)	3561 ± 643	1023 ± 771	**0.003**	4699 ± 843	1957 ± 301	**< 0.001**
LVEF (%)	21.98 ± 5.91	41.21 ± 8.37	**< 0.001**	23.25 ± 9.09	31.44 ± 11.51	**< 0.001**
NYHA III	102 (82%)	0 (0%)	**< 0.001**	58 (85%)	51 (43%)	0.175
Hypertension	85 (69%)	37 (59%)	**0.197**	49 (72%)	73 (61%)	0.154
Diabetes	31 (25%)	8 (13%)	0.058	16 (24%)	23 (19%)	0.575
Atrial fibrillation	55 (44%)	26 (41%)	0.756	29 (43%)	52 (44%)	1.000
CKD	46 (37%)	15 (24%)	0.072	29 (43%)	32 (27%)	**0.035**
ICD	48 (39%)	32 (51%)	**0.049**	31 (67%)	49 (41%)	0.645
CRT‐D	65 (52%)	25 (40%)	0.559	27 (40%)	63 (53%)	0.095
Beta blocker	103 (83%)	56 (89%)	0.387	50 (74%)	'109 (92%)	**0.001**
Amiodarone	78 (63%)	25 (40%)	**0.003**	49 (72%)	54 (45%)	**< 0.001**
Prior VA ablation	38 (31%)	16 (25%)	0.319	19 (28%)	35 (29%)	0.868
PAINESD score	13.03 ± 07.08	11.35 ± 6.91	**< 0.001**	17.68 ± 5.67	11.94 ± 6.93	**< 0.001**

*Note:* A *p*‐value < 0.05 indicates statistical significance (bold).

Abbreviations: CKD, chronic kidney disease; CRT‐D, cardiac resynchronization therapy; ECMO, extracorporeal membrane oxygenation; HS, hemodynamic support; ICD, implantable cardioverter defibrillator; ICM, ischemic cardiomyopathy; LVAD, left ventricular assist device; LVEF, left ventricular ejection fraction; NICM, nonischemic cardiomyopathy; NYHA, New York Heart Association functional class; VA, ventricular arrhythmia.

#### 3.2.2 No HS Versus HS (Impella/ECMO/LVAD)

**Figure 2:**
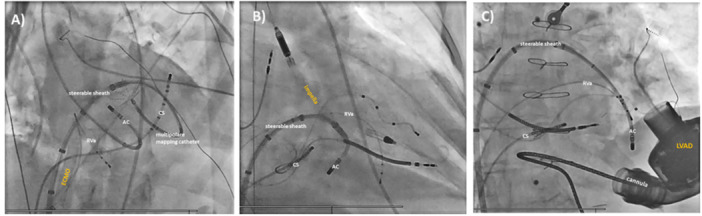
Representative images of ventricular arrhythmia ablation with HS with (A) ECMO, (B) Impella, and (C) LVAD. AC, ablation catheter; CS, coronary sinus; ECMO, extracorporeal membrane oxygenation; HS, hemodynamic support; LVAD, left ventricular assist device; RVa, right ventricle apex; VA, ventricular arrhythmia.

68/119 patients (36%) received VA ablation with HS (mean age 60.8 ± 9.8 years, 16% female). These patients had substantially higher NT‐proBNP values, a lower LVEF, a more frequent AAD therapy with Amiodarone, and higher PAINESD‐Score values (Table 1). Baseline characteristics of patients with different types of HS are gathered in Table 2. In Figure 2 fluoroscopy images of VA ablation using different types of HS are presented.

**Table 2:** Baseline characteristics (ECMO/Impella/LVAD).

Characteristics	ECMO (*n* = 20)	Impella (*n* = 19)	LVAD (*n* = 29)	*p*‐value no HS (I + II) versus ECMO	*p*‐value no HS (I + II) versus Impella	*p*‐value no HS (I + II) versus LVAD
Age (years)	59.35 ± 11.37	61.79 ± 11.21	61.10 ± 7.66	0.356	0.957	0.636
Gender, Female	5 (25%)	2 (11%)	4 (14%)	0.152	1.000	0.755
ICM	11 (55%)	14 (74%)	19 (66%)	0.812	0.085	0.214
NICM	9 (45%)	5 (26%)	10 (44%)	0.812	0.085	0.214
NTproBNP (pg/mL)	4699 ± 843	1957 ± 301	1957 ± 301	0.408	0.456	0.505
LVEF (%)	26.20 ± 11.52	24.00 ± 11.21	20.72 ± 7.45	**0.049**	**0.005**	**< 0.001**
NYHA III	16 (80%)	19 (100%)	28 (97%)	**0.003**	**< 0.001**	**< 0.001**
Hypertension	12 (60%)	15 (79%)	21 (72%)	1.000	0.132	0.287
Diabetes	5 (25%)	4 (21%)	7 (24%)	0.554	0.766	0.130
Atrial fibrillation	6 (30%)	8 (42%)	14 (48%)	0.329	1.000	0.682
CKD	4 (20%)	7 (37%)	17 (59%)	0.594	0.414	**< 0.001**
ICD	7 (35%)	11 (58%)	13 (45%)	0.806	0.215	0.673
CRT‐D	6 (30%)	5 (26%)	15 (52%)	0.089	**0.046**	0.839
Beta blocker	14 (70%)	14 (74%)	21 (72%)	**0.013**	**0.035**	**0.009**
Amiodarone	15 (75%)	14 (74%)	19 (66%)	**0.016**	**0.027**	0.063
Prior VA ablation	12 (60%)	10 (53%)	15 (52%)	**0.012**	0.064	**0.029**
PAINESD score	16.80 ± 6.56	17.26 ± 5.60	18.55 ± 5.03	**0.006**	**< 0.001**	**< 0.001**

*Note:* A *p*‐value < 0.05 indicates statistical significance (bold).

Abbreviations: CKD, chronic kidney disease; CRT‐D, cardiac resynchronization therapy; ECMO, extracorporeal membrane oxygenation; HS, hemodynamic support; ICD, implantable cardioverter defibrillator; ICM, ischemic cardiomyopathy; LVAD, left ventricular assist device; LVEF, left ventricular ejection fraction; NICM, nonischemic cardiomyopathy; NYHA, New York Heart Association functional class; VA, ventricular arrhythmia.

#### 3.2.3 Procedural Data

In patients who had required HS, longer fluoroscopy times were observed. In advanced HF patients and in patients with HS a significantly higher number of VAs was documented. VA inducibility was lower in advanced HF patients compared to patients without advanced HF and even lower in advanced HF patients that underwent VA ablation procedures with HS (Tables 3, 4, and S2).

**Table 3:** Procedural data (advanced HF vs. no advanced HF, HS vs. no HS).

Characteristics	Advanced HF (I + III) (*n* = 124)	No advanced HF (II + IV) (*n* = 63)	*p*‐value	HS (III + IV) (*n* = 68)	No HS (I + II) (*n* = 119)	*p*‐value
Procedure Duration (min)	215.28 ± 63.97	218.89 ± 6.90	0.724	213.39 ± 70.80	218.15 ± 63.92	0.649
Fluoroscopy Time (min:s)	8:26 ± 5:70	6:54 ± 6:55	0.194	10:06 ± 6:56	6:56 ± 5:35	**< 0.001**
Fluoroscopy Dose (yGmy^2^)	1412.62 ± 253.63	1278.21 ± 215.18	0,705	1811.45 ± 291.23	1118.04 ± 202.24	0.078
Mapping and Ablation						
Endocardial	104 (84%)	50 (79%)	0.543	58 (85%)	96 (81%)	0.550
Endocardial + Epicardial	19 (15%)	13 (21%)	0.413	9 (13%)	23 (19%)	0.319
Left ventricle	103 (83%)	49 (78%)	0.429	51 (75%)	101 (85%)	0.119
Right ventricle	3 (2%)	1 (2%)	1.000	4 (6%)	0 (0%)	**0.017**
Left ventricle + Right ventricle	18 (15%)	13 (21%)	0.304	13 (19%)	18 (15%)	0.541
Number of VAs	2.05 ± 2.00	1.40 ± 0.90	**0.024**	2.25. ± 2.18	1.40 ± 0.90	**< 0.001**
VA inducibility before ablation	99 (80%)	58 (92%)	**0.035**	49 (72%)	108 (91%)	**0.002**
Substrate modification	105 (85%)	54 (86%)	1.000	54 (79%)	105 (88%)	0.135

*Note:* A *p*‐value < 0.05 indicates statistical significance (bold).

Abbreviations: HF, heart failure; HS, hemodynamic support.

**Table 4:** Procedural data (ECMO/Impella/LVAD).

Characteristics	ECMO (*n* = 20)	Impella (*n* = 19)	LVAD (*n* = 29)	*p*‐value no HS (I + II) versus ECMO	*p*‐value no HS (I + II) versus Impella	*p*‐value no HS (I + II) versus LVAD
Procedure Duration (min)	205.50 ± 82.05	237.95 ± 61.44	202.35 ± 66.35	**< 0.001**	**< 0.001**	**< 0.001**
Fluoroscopy time (min:s)	07:19 ± 3:30	9:58 ± 7:31	12:18 ± 7:53	**< 0.001**	**< 0.001**	**< 0.001**
Fluoroscopy dose (yGmy^2^)	1948.45 ± 341.78	2335.99 ± 366.86	1378.03 ± 182.46	0.233	0.173	0.505
Mapping and ablation						
Endocardial	16 (80%)	14 (74%)	28 (97%)	1.000	0.539	**0.047**
Endocardial + Epicardial	4 (20%)	4 (21%)	1 (3%)	1.000	0.766	**0.047**
Left ventricle	16 (80%)	15 (79%)	20 (69%)	0.524	0.506	0.104
Right ventricle	2 (10%)	1 (5%)	1 (3%)	**0.019**	0.138	0.196
Left ventricle + Right ventricle	2 (10%)	3 (16%)	8 (28%)	0.738	1.000	0.170
Number of VAs	2.30 ± 2.60	2.30 ± 2.44	2.34 ± 1.84	**< 0.001**	**< 0.001**	**< 0.001**
VA inducibility before ablation	18 (90%)	18 (95%)	27 (93%)	1.000	1.000	1.000
Substrate modification	16 (%)	17 (%)	28 (97%)	0.295	1.000	0.305

*Note:* A *p*‐value < 0.05 indicates statistical significance (bold).

Abbreviations: ECMO, extracorporeal membrane oxygenation; HF, heart failure; HS, hemodynamic support; LVAD, left ventricular assist device; VA, ventricular arrhythmia.

#### 3.2.4 Acute Success, Complications, and In‐Hospital Mortality

Concerning acute procedural success and major complications, no significant differences were observed between groups (Tables 5 and S3). Major procedural complications included bleeding (*n* = 3), pericardial tamponade or effusion requiring drainage (*n* = 2), stroke (*n* = 2), and vascular complications such as access site hematoma or arteriovenous fistula requiring intervention (*n* = 2).

**Table 5:** Acute success, complications, and in hospital mortality (advanced HF vs. no advanced HF, HS vs. no HS).

Characteristics	Advanced HF (I + III) (*n* = 124)	No advanced HF (II + IV) (*n* = 63)	*p*‐value	HS (III + IV) (*n* = 68)	No HS (I + II) (*n* = 119)	*p*‐value
Acute procedural success	118 (95%)	63 (100%)	0.099	65 (96%)	116 (97%)	0.679
Major procedural complications	5 (4%)	4 (6%)	0.488	4 (6%)	5 (4%)	0.726
Procedure associated death	0 (0%)	0 (0%)	1.000	0 (0%)	0 (0%)	1.000
In Hospital Mortality	19 (15%)	2 (3%)	**< 0.001**	20 (29%)	1 (1%)	**< 0.001**
Time to death (days) (min, max)	20 (2, 362)	4.5 (4, 5)		19 (2, 362)	4 (‐, ‐)	
Cause of death and timepoint						
Cardiogenic shock (days)	7 (2, 5, 14, 18, 29, 29, 49)	2 (4, 5)		8 (2, 2, 5, 14, 18, 29, 29, 49)	1 (4)	
Multi‐organ failure (days)	9 (3, 7, 8, 16, 18, 22, 22, 56, 362)	—		9 (3, 7, 8, 16, 18, 22, 22, 56, 362)	—	
Sepsis (days)	2 (55, 63)	—		2 (55, 63)	—	
Stroke (days)	1 (23)	—		1 (23)	—	

*Note:* A *p*‐value < 0.05 indicates statistical significance (bold).

Abbreviations: HF, heart failure; HS, hemodynamic support.

In patients with advanced HF and especially in the HS cohort of patients (ECMO and LVAD)—except for patients with an Impella—significantly higher in‐hospital mortality rates were observed compared to patients without advanced HF and without the need for HS. Importantly, no deaths were procedure related. In‐hospital mortality was predominantly driven by cardiogenic shock and multi‐organ failure. The temporal relationship between ablation and death showed no evidence of ablation‐related clinical deterioration (Tables 5 and 6).

**Table 6:** Acute success, complications, and in hospital mortality (ECMO/Impella/LVAD).

Characteristics	ECMO (*n* = 20)	Impella (*n* = 19)	LVAD (*n* = 29)	*p*‐value no HS (I + II) versus ECMO	*p*‐value no HS (I + II) versus Impella	*p*‐value no HS (I + II) versus LVAD
Acute procedural success	19 (95%)	17 (89%)	29 (100%)	0.467	0.139	1.000
Major procedural complications	2 (10%)	1 (5%)	1 (3%)	0.265	1.000	1.000
Procedure associated death	0 (0%)	0 (0%)	0 (0%)	1.000	1.000	1.000
In hospital mortality	10 (50%)	2 (11%)	8 (28%)	**< 0.001**	0.050	**< 0.001**
Time to death (days, min, max)	'18 (3, 63)	17 (5, 29)	22 (2, 362)			
Cause of death + timepoint						
Cardiogenic shock (days)	4 (14, 18, 29, 49)	2 (5, 29)	2 (2, 2)			
Multi‐organ failure (days)	4 (3, 7, 8, 18)	—	5 (16, 22, 22, 56, 362)			
Sepsis (days)	1 (63)	—	1 (55)			
Stroke (days)	1 (23)	—	—			

*Note:* A *p*‐value < 0.05 indicates statistical significance (bold).

Abbreviations: ECMO, extracorporeal membrane oxygenation; HF, heart failure; HS, hemodynamic support; LVAD, left ventricular assist device; VA, ventricular arrhythmia.

#### 3.2.5 24‐month VA‐Free Survival and Mortality

**Figure 3:**
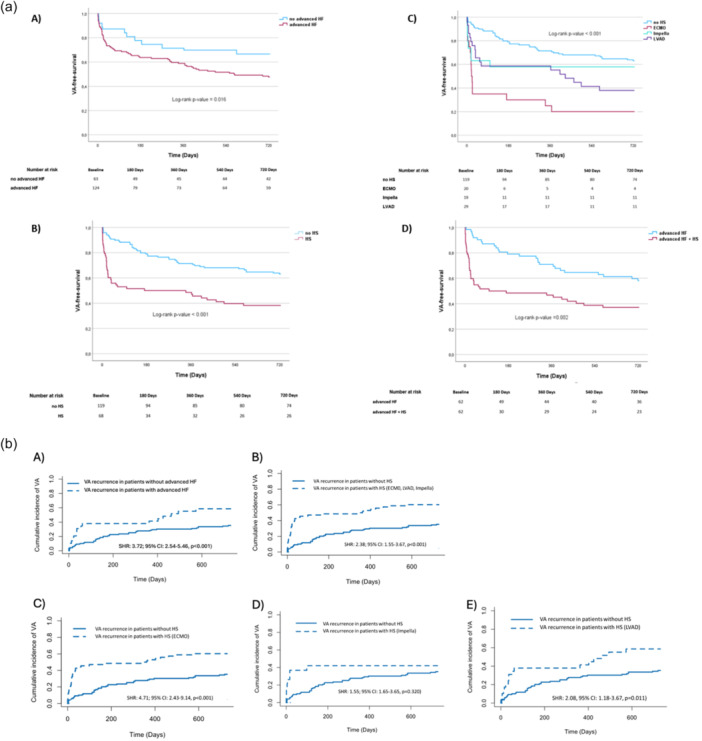
(a) Kaplan–Meier plot analyses on 24‐month VA‐free survival. (A) Patients with advanced HF presented with a significantly lower VA‐free survival rate compared to those without. (B) Patients with pre‐emptive HS showed a higher VA‐recurrence rate compared to the group of patients without HS. (C) HS with an ECMO and LVAD, but not with an Impella, was associated with a significantly lower VA‐free survival rate in contrast to patients without HS. (D) Subgroup analyses of patients with advanced HF compared to those with advanced HF that had required HS additionally, revealed a significantly higher VA‐recurrence rate in the latter. (b) CIF of VA recurrence accounting for the competing risk of death, analyzed using Fine and Gray SHR. Advanced HF (A), pre‐emptive HS (B), and HS with an ECMO (C) or LVAD (D) were associated with a significantly increased risk of VA recurrence, whereas Impella support (E) was not associated with a statistically significant increase in VA recurrence. CIF, cumulative incidence function; ECMO, extracorporeal membrane oxygenation; HF, heart failure; HS, hemodynamic support; LVAD, left ventricular assist device; SHR, subdistribution hazard model; VA, ventricular arrhythmia.

Kaplan–Meier plot analyses revealed a lower estimated VA‐free survival rate and a higher mortality in patients with advanced HF compared to those without (Figures 3aA and 4A). The need for HS with an ECMO and a LVAD was associated with an even higher VA‐recurrence rate and mortality (Figures 3aB,C and 4B,C) whereas no significant differences were observed between patients without HS and patients with an Impella (Figures 3aC and 4C). Even when death is considered as a competing risk, there remains a strong and statistically significant association between advanced HF (Figure 3bA), pre‐emptive HS (Figure 3bB), and HS with ECMO (Figure 3bC) or LVAD (Figure 3bD) and an increased risk of VA recurrence. In contrast, Impella support (Figure 3bE) did not demonstrate a significant association of elevated VA recurrence.

**Figure 4:**
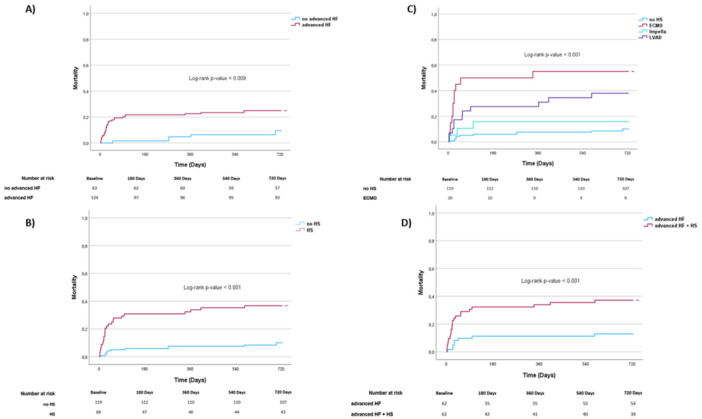
Kaplan–Meier plot analyses on 24‐month mortality. (A) Patients with advanced HF presented with a significantly higher mortality rate compared to those without. (B) Patients with pre‐emptive HS showed a higher mortality compared to the group of patients without HS. (C) HS with an ECMO and LVAD, but not with an Impella, was associated with a significantly higher mortality in contrast to patients without HS. (D) Subgroup analyses of patients with advanced HF compared to those with advanced HF that had required HS additionally, revealed a significantly higher mortality in the latter. ECMO, extracorporeal membrane oxygenation; HF, heart failure; HS, hemodynamic support; LVAD, left ventricular assist device; VA, ventricular arrhythmia.

Subgroup analyses showed that advanced HF patients requiring HS had lower VA‐free survival and higher mortality rates compared with advanced HF patients not requiring HS. (Figures 3aD and 4D). Three patients initially treated with an ECMO received an LVAD during follow‐up. Four patients—two of them initially treated with an Impella, one with an ECMO and one with a LVAD—received a heart transplantation within the observation period.

Cox regression model analyses revealed that HS with an ECMO was associated with the highest risk of both VA recurrence and mortality. LVAD support was significantly associated with increased VA recurrence and showed a borderline association with mortality. In contrast, pre‐emptive HS using an Impella was not significantly associated with either outcome (Table S4).

## 4 Discussion

This observational study has three major findings: First, even in the advanced HF and HS cohort of patients VA ablation procedures seem to be safe and effective with comparable acute success and complication rates. Second, the need for HS seems to be associated with a worse 24‐month VA‐free survival and mortality rate (in hospital, and over 24 months). This applies in particular to patients that require HS with an ECMO and LVAD. Third, VA‐recurrence and mortality rates in patients with an Impella do not differ significantly from those who did not require HS.

### 4.1 Ablation of VA in Patients With Advanced HF

Advanced HF patients had multiple comorbidities (Table 1) and a substantially higher number of VA (Table 2), which may be attributed to a larger amount of scarred tissue. Consecutively, 24‐month freedom from VA recurrence (Figure 3aA) and mortality (Figure 4A) following ablation without HS was significantly different between patients with advanced HF and those without.

This underscores the significance of a precise preprocedural risk assessment, including the PAINESD score and preprocedural imaging [13, 14]. Adherent to the current ESC‐guidelines, CMR is recommend for the decision‐making of individuals with arrhythmic events [15]. As arrhythmogenic substrates detected on late gadolinium enhancement (LGE) cardiac magnetic resonance imaging (CMR) can provide prognostic value to major cardiac events and mortality [16, 17] including the amount of scar tissue into the PAINESD score may be beneficial [18]. Beyond that, the novel inHEART analysis approach (IHU Liryc, Pessac, France), which is based on a LGE CT‐image morphological assessment of the ventricles, has the potential to visualize VA substrate beyond conventional electrogram‐based mapping and thus may help to further increase the rate of successful VA ablation procedures [19]. Given the bidirectional causality between increased VA burden and accelerated disease progression and mortality [20], it may be beneficial to schedule these patients for VA ablation procedures at an earlier stage of their disease, in accordance with recent randomized trials that demonstrate the advantages of early CA of VA [21, 22, 23, 24].

### 4.2 Relationship Between HS, Freedom From Arrhythmia Recurrence, and Mortality

Concerning acute procedural success (97%) and severe adverse events (2%–6%) no significant group differences were observed (Tables 5, 6, and S3). This may be partially explained by the pre‐emptive use of HS and a comprehensive preprocedural risk assessment. Lower acute success and higher complication rates in prior studies [9, 25, 26] may be attributed to the use of HS as a rescue tool or its utilization predominantly in very sick patients. In addition, the need of anticoagulation may be associated with complications in patients with HS devices, too [9].

In‐hospital mortality rates (Tables 5, 6, and S3) were comparable to those reported in previous studies [9]. Importantly, no in‐hospital mortality was considered procedure‐related, and no evidence of procedure‐induced clinical deterioration was observed. These findings suggest that in‐hospital mortality in this cohort primarily reflects advanced disease severity and systemic deterioration within this high‐risk population rather than complications directly related to the ablation procedure.

Patients who received ablation with HS had a significantly increased estimated 24‐month VA‐recurrence (Figure 3aB) and mortality (Figure 4B). This remains true even when considering death as a competing risk (Figure 3bB).

This is consistent with previous studies, which demonstrated that there is no significant benefit in terms of VA‐free survival and mortality, despite the improved ability to perform VA mapping and ablation [25, 26, 27, 28]. However, the majority of studies did not differentiate between various types of HS, despite the fact that the specific form of HS is essential for the interpretation of study results and patients’ outcome.

### 4.3 Impact of the Type of HS on Freedom From Arrhythmia Recurrence and Mortality

A few smaller retrospective studies in patients who received ablation with Impella reported a modest reduction in VA‐recurrence rates [25, 27, 29], but a markedly higher mortality rate [27]. In our study, mortality was not significantly increased (Table 3) which may be attributed to the fact that Impella devices were not implanted as a rescue tool in a bailout situation.

Previous studies in patients with ECMO‐support to guide ablation documented a 23‐month VA‐free survival rate of 88% [29, 30, 31, 32]. Our results are consistent with these findings (Figure 3aC). However, ECMO patients exhibited a significantly lower survival rate compared to patients without HS (Figure 4C). The main rationale for this observation is that ECMO implantation was restricted to high‐risk patients with a preprocedural AHD.

In a pooled analysis of multiple LVAD‐VT case series, a 9‐month‐VA‐free survival rate of 56% was documented [12]. This is consistent with our findings, too (Figure 3aC). The main reason of VA in end‐stage HF and LVAD is the underlying myocardial arrhythmia substrate, although it may also be attributed to the LVAD itself [12, 33]. In contrast to the pooled analysis, our study showed a lower mortality rate of only 38% instead of 48% (Figure 4C).

Multivariate analyses identified HS as an independent predictor of VA‐recurrence and mortality in patients with an ECMO and LVAD, but not with an Impella (Table S4). This may be mainly attributed to various clinical scenarios. Whereas serious comorbidities and disease progression may be the primary factors contributing to a worse outcome in critical ill patients requiring HS with an ECMO or an LVAD, pre‐emptive HS with an Impella appears to enable advanced HF patients to achieve outcomes that are comparable to those of patients in better health conditions. This indicates that a comprehensive pre‐procedural risk assessment regarding the pre‐emptive use of an Impella for VA ablation procedures is essential to enhance VA‐free survival and mortality in high‐risk patients.

## 5 Limitations

Our study is a retrospective observational study with the inherent limitations. The cohort of patients requiring HS was small. This study includes both temporary (ECMO, Impella) and permanent HS (LVAD). These modalities represent distinct clinical entities with different hemodynamic profiles, arrhythmia substrates and long‐term disease trajectories, which may limit direct comparability. Although subgroup analyses were performed, residual confounding cannot be excluded. A systematic and standardized assessment of the RV function was not available in all patients. Right heart catheterization to guide the selection of HS was not routinely performed prior to ablation. In addition, echocardiographic RV assessment was also not consistently available in all patients. Prospective trials are warranted to analyze the impact of different types of HS on patients’ VA‐free survival and mortality in more detail.

## 6 Conclusion

In high‐risk HF patients, VA ablation was feasible with comparable acute success and procedural complication rates. Patients supported with ECMO or LVAD showed higher mortality and VA recurrence rates compared to Impella‐supported procedures, likely reflecting more advanced disease severity rather than differences in procedural efficacy.

## Funding

The authors have nothing to report.

## Conflicts of Interest

Christian Sohns received research support and lecture fees from Medtronic, Abbott, Boston Scientific, and J&J MedTec; is a consultant for Medtronic, Boston Scientific, and J&J MedTec; has received grant support from the Else Kröner‐Fresenius‐Stiftung and Deutsche Herzstiftung. Philipp Sommer is Member of Advisory Board for Abbott, Boston Scientific, J&J MedTech and Medtronic. The remaining authors declare no conflicts of interest.

## Supporting information


**Table S1:** Baseline characteristics (Groups I‐IV). CKD, chronic kidney disease; CRT‐D, cardiac resynchronization therapy; HS, hemodynamic support; ICD, implantable cardioverter defibrillator; ICM, ischemic cardiomyopathy; LVEF, left ventricular ejection fraction; NICM, nonischemic cardiomyopathy; NYHA, New York Heart Association functional class; VA, ventricular arrhythmia. A *p*‐value < 0.05 indicates statistical significance;
**Table S2:** Procedural Data (Groups I‐IV). HF, heart failure; HS, hemodynamic support; VA, ventricular arrhythmia. A *p*‐value < 0.05 indicates statistical significance;
**Table S3:** Acute Success and Complications (Groups I‐IV). HF, heart failure; HS, hemodynamic support; VA, ventricular arrhythmia. A *p*‐value < 0.05 indicates statistical significance;
**Table S4:** Cox regression analyses. ECMO, extracorporeal membrane oxygenation; HF, heart failure; HS, hemodynamic support; LVAD, Left ventricular assist device. A *p*‐value < 0.05 indicates statistical significance.

## Data Availability

The data that support the findings of this study are available from the corresponding author upon reasonable request.
