# Supplementary material for: Impacts of Hemodynamic Support on Ventricular Arrhythmia Ablation
Source: J Cardiovasc Electrophysiol. 2026 Jun 9;37(8):1711–22. doi: 10.1111/jce.70396 (PMC13475177; doi:10.1111/jce.70396)
Supplement: Supplementary file 1 [file JCE-37-1711-s001.pdf]

Supplemental Table 1

Characteristics	Advanced HF without HS (I) (n=62)	No Advanced HF without HS (II) (n=57)	Advanced HF with HS (III) (n=62)	No advanced HF with HS (IV) (n=6)	p-value (I vs. II)	p-value (III vs. IV)	p-value (I vs. III)	p-value (I+II vs. III+IV)
**Age (years)**	63.47 ± 8.91	60.28 ± 13.80	60.65 ± 9.29	62.17 ± 14.92	0.142	0.815	0.087	0.466
**Gender, Female**	7 (11%)	7 (12%)	10 (16%)	1 (17%)	1.000	1.000	0.603	0.503
**ICM**	40 (65%)	21 (37%)	39 (63%)	5 (83%)	**0.003**	0.413	1.000	0.092
**NICM**	22 (35%)	36 (63%)	23 (37%)	1 (17%)	**0.003**	0.413	1.000	0.092
**NTproBNP (pg/mL)**	2486 ± 301	432 ± 57	4977 ± 681	1034 ± 639	**0.002**	**0.037**	0.176	0.112
**LVEF (%)**	22.82 ± 5.57	40.81 ± 8.61	21.15 ± 6.26	45.00 ± 2.98	**<0.001**	**<0.001**	0.114	**<0.001**
**NYHA III**	51 (82%)	0 (0%)	58 (94%)	0 (0%)	**<0.001**	**<0.001**	0.096	**<0.001**
**Hypertension**	41 (66%)	32 (56%)	44 (71%)	5 (83%)	0.346	1.000	0.699	0.154
**Diabetes**	17 (27%)	6 (11%)	14 (23%)	2 (33%)	**0.022**	0.620	0.679	0.575
**Atrial fibrillation**	31 (50%)	22 (39%)	25 (40%)	4 (67%)	0.268	0.389	0.367	0.879
**CKD**	18 (29%)	14 (25%)	28 (45%)	1 (17%)	0.680	0.229	0.094	**0.035**
**ICD**	21 (34%)	28 (49%)	27 (44%)	4 (67%)	0.098	0.400	0.357	0.645
**CRT-D**	39 (63%)	24 (42%)	26 (42%)	1 (17%)	**0.028**	0.390	**0.031**	0.095
**Beta Blocker**	59 (95%)	50 (88%)	44 (71%)	6 (100%)	0.192	0.184	**<0.001**	**0.001**
**Amiodarone**	33 (53%)	21 (37%)	45 (73%)	4 (67%)	0.097	0.669	**0.025**	**<0.001**
**Prior VA Ablation**	22 (67%)	13 (23%)	16 (26%)	3 (50%)	**0.028**	0.338	0.330	0.868
**PAINESD Score**	13.03 ± 7.08	8.09 ± 5.21	17.74 ± 5.51	17.00 ± 7.69	**<0.001**	0.826	**<0.001**	**<0.001**

Supplemental Table 2

Characteristics	Advanced HF without HS (I) (n=62)	No Advanced HF without HS (II) (n=57)	Advanced HF with HS (III) (n=62)	No advanced HF with HS (IV) (n=6)	p-value (I vs. II)	p-value (III vs. IV)	p-value (I vs. III)	p-value (I+II vs. III+IV)
**Procedure Duration (min)**	217.77 ± 56.19	218.56 ± 71.90	220.00 ± 14.49	212.74 ± 71.40	0.947	**<0.001**	0.664	0.067
**Fluoroscopy Time (min:s)**	6:39 ± 3:00	6:54 ± 7:10	10:12 ± 7:20	9:22 ± 2:57	0.732	0.559	**<0.001**	**0.019**
**Fluoroscopy Dose (yGmy^2)**	946.65 ± 180.49	1180.92 ± 214.62	1878.59 ± 303.50	978.96 ± 187.29	0.531	0.066	**0.039**	0.067
**Mapping and Ablation**								
**Endocardial**	50 (81%)	46 (81%)	54 (87%)	4 (67%)	0.822	0.212	0.465	0.550
**Endocardial + Epicardial**	12 (19%)	11 (19%)	7 (11%)	2 (33%)	1.000	0.177	0.319	0.319
**Left ventricle**	55 (89%)	46 (81%)	48 (77%)	3 (50%)	0.307	0.160	0.150	0.119
**Right ventricle**	0 (0%)	0 (0%)	3 (4%)	1 (17%)	1.000	0.315	0.244	**0.017**
**Left + Right Ventricle**	7 (11%)	11 (19%)	11 (18%)	2 (33%)	0.307	0.322	0.445	0.541
**Number of VAs**	2.00 ± 1.00	1.40 ± 0.90	2.21 ± 2.23	2.67 ± 1.75	**0.024**	0.572	0.644	0.094
**VA inducibilty before ablation**	56 (90%)	52 (91%)	43 (69%)	6 (100%)	1.000	0.175	**0.006**	**0.002**
**Substrate modification**	57 (92%)	48 (84%)	48 (77%)	6 (100%)	0.257	0.333	**0,044**	0.135

Supplemental Table 3

Characteristics	Advanced HF without HS (I) (n=62)	No Advanced HF without HS (II) (n=57)	Advanced HF with HS (III) (n=62)	No advanced HF with HS (IV) (n=6)	p-value (I vs. II)	p-value (III vs. IV)	p-value (I vs. III)	p-value (I+II vs. III+IV)
**Acute success**	59 (95%)	57 (100%)	59 (95%)	6 (100%)	0.245	1.000	1.000	0.669
**Major complications**	1 (2%)	4 (7%)	4 (6%)	0 (0%)	0.193	1.000	0.365	0.726
**In Hospital Mortality**	1 (2%)	0 (0%)	18 (29%)	2 (33%)	1.000	1.000	**<0.001**	**<0.001**

Supplemental Table 4

	VA-recurrence			Mortality		
**Parameter**	**Hazard ratio**	**Confidence interval**	**p-value**	**Hazard ratio**	**Confidence interval**	**p-value**
**HS (ECMO)**	9.78	3.228 - 19.614	**<0.001**	5.76	3.175 - 18.230	**0.031**
**HS (Impella)**	1.27	0.291 - 3.548	0.749	2.24	0.946 – 10.337	0.475
**HS (LVAD)**	5.17	2.002 - 13.363	**<0.001**	4.08	1.967 - 7.190	0.056
**NTproBNP**	1.00	1.000 - 1.000	0.155	1.00	1.000 - 1.000	0.218
**PAINESD Score**	0.94	0.885 – 1.000	0.051	0.97	0.886 - 1.063	0.520
